# Supplementary material for: The effects of resveratrol feeding and exercise training on the skeletal muscle function and transcriptome of aged rats
Source: PeerJ. 2019 Jul 1;7:e7199. doi: 10.7717/peerj.7199 (PMC6610545; doi:10.7717/peerj.7199)
Supplement: Table S1 — Old: old rat; Trained: old rat treated by exercise training; Resveratrol: old rat treated by oral resveratrol; a, b, c: Three replicate samples of each treatment. [file peerj-07-7199-s001.doc]

Table S1 Summary of the raw sequencing data of the gastrocnemius muscle tissue of the rats treated with six weeks of exercise training and resveratrol feeding compared to the control rats.

| Sample name | Raw reads | Clean reads | clean bases | Error rate (%) | Q20 (%) | Q30 (%) | GC content (%) | Mapped reads | Mapping ratio (%) |
| --- | --- | --- | --- | --- | --- | --- | --- | --- | --- |
| Old-a | 70121916 | 68299998 | 10.24G | 0.03 | 96.64 | 94.41 | 51.07 | 62420988 | 91.4 |
| Old-b | 60967736 | 59707178 | 8.96G | 0.02 | 97.55 | 95.9 | 51.02 | 55740250 | 93.4 |
| Old-c | 62900766 | 61950172 | 9.29G | 0.02 | 97.46 | 95.76 | 50.31 | 57735348 | 93.2 |
| Trained-a | 78188802 | 76550156 | 11.48G | 0.03 | 96.63 | 94.32 | 50.99 | 71430114 | 93.3 |
| Trained-b | 82621676 | 81018930 | 12.15G | 0.03 | 96.64 | 94.33 | 50.55 | 75568582 | 93.3 |
| Trained-c | 56658316 | 55811154 | 8.37G | 0.02 | 97.56 | 95.92 | 51.42 | 52165064 | 93.5 |
| Resveratrol-a | 83259734 | 81891396 | 12.28G | 0.03 | 96.61 | 94.3 | 50.85 | 76365653 | 93.3 |
| Resveratrol-b | 91595178 | 89760972 | 13.46G | 0.03 | 96.56 | 94.24 | 49.86 | 83514362 | 93.0 |
| Resveratrol-c | 84324858 | 82618126 | 12.39G | 0.03 | 96.48 | 94.13 | 51.1 | 76837133 | 93.0 |
