# Supplementary material for: The effects of resveratrol feeding and exercise training on the skeletal muscle function and transcriptome of aged rats
Source: PeerJ. 2019 Jul 1;7:e7199. doi: 10.7717/peerj.7199 (PMC6610545; doi:10.7717/peerj.7199)
Supplement: Table S2 — Old: old rat; Trained: old rat treated by exercise training; GO: Gene Ontology [file peerj-07-7199-s002.doc]

Table S2 The significant Gene Ontology terms of the rats treated with six weeks of exercise training compared to the control rats.

| GO accession | Description | Term type | Over represented p Value | DEG item | Up | Down |
| --- | --- | --- | --- | --- | --- | --- |
| GO:0032545 | CURI complex | cellular_component | 0.001121 | 1 | 0 | 1 |
| GO:0034456 | UTP-C complex | cellular_component | 0.001121 | 1 | 0 | 1 |
| GO:0033553 | rDNA heterochromatin | cellular_component | 0.001899 | 1 | 0 | 1 |
| GO:0071217 | cellular response to external biotic stimulus | biological_process | 0.002067 | 1 | 1 | 0 |
| GO:0004126 | cytidine deaminase activity | molecular_function | 0.002102 | 1 | 1 | 0 |
| GO:0006216 | cytidine catabolic process | biological_process | 0.002102 | 1 | 1 | 0 |
| GO:0009972 | cytidine deamination | biological_process | 0.002102 | 1 | 1 | 0 |
| GO:0046087 | cytidine metabolic process | biological_process | 0.002102 | 1 | 1 | 0 |
| GO:0034660 | ncRNA metabolic process | biological_process | 0.002697 | 3 | 0 | 3 |
| GO:0046133 | pyrimidine ribonucleoside catabolic process | biological_process | 0.003317 | 1 | 1 | 0 |
| GO:0046015 | regulation of transcription by glucose | biological_process | 0.003792 | 1 | 0 | 1 |
| GO:0000183 | chromatin silencing at rDNA | biological_process | 0.004144 | 1 | 0 | 1 |
| GO:0046135 | pyrimidine nucleoside catabolic process | biological_process | 0.004529 | 1 | 1 | 0 |
| GO:0046898 | response to cycloheximide | biological_process | 0.005478 | 1 | 1 | 0 |
| GO:0006364 | rRNA processing | biological_process | 0.005946 | 2 | 0 | 2 |
| GO:0016072 | rRNA metabolic process | biological_process | 0.00631 | 2 | 0 | 2 |
| GO:0004653 | polypeptide N-acetylgalactosaminyltransferase activity | molecular_function | 0.007623 | 1 | 1 | 0 |
| GO:0005677 | chromatin silencing complex | cellular_component | 0.008877 | 1 | 0 | 1 |
| GO:0000813 | ESCRT I complex | cellular_component | 0.009674 | 1 | 0 | 1 |
| GO:0005375 | copper ion transmembrane transporter activity | molecular_function | 0.010768 | 1 | 0 | 1 |
| GO:0035434 | copper ion transmembrane transport | biological_process | 0.010768 | 1 | 0 | 1 |
| GO:0032509 | endosome transport via multivesicular body sorting pathway | biological_process | 0.012058 | 1 | 0 | 1 |
| GO:0015269 | calcium-activated potassium channel activity | molecular_function | 0.012472 | 1 | 0 | 1 |
| GO:0042454 | ribonucleoside catabolic process | biological_process | 0.013826 | 1 | 1 | 0 |
| GO:0036452 | ESCRT complex | cellular_component | 0.014108 | 1 | 0 | 1 |
| GO:0019835 | cytolysis | biological_process | 0.014289 | 1 | 0 | 1 |
| GO:0072529 | pyrimidine-containing compound catabolic process | biological_process | 0.014616 | 1 | 1 | 0 |
| GO:0009164 | nucleoside catabolic process | biological_process | 0.015582 | 1 | 1 | 0 |
| GO:0009303 | rRNA transcription | biological_process | 0.016305 | 1 | 0 | 1 |
| GO:0042254 | ribosome biogenesis | biological_process | 0.0165 | 2 | 0 | 2 |
| GO:0008376 | acetylgalactosaminyltransferase activity | molecular_function | 0.016551 | 1 | 1 | 0 |
| GO:0046131 | pyrimidine ribonucleoside metabolic process | biological_process | 0.016909 | 1 | 1 | 0 |
| GO:0019239 | deaminase activity | molecular_function | 0.017527 | 1 | 1 | 0 |
| GO:1901658 | glycosyl compound catabolic process | biological_process | 0.017894 | 1 | 1 | 0 |
| GO:0034470 | ncRNA processing | biological_process | 0.017902 | 2 | 0 | 2 |
| GO:0016814 | hydrolase activity, acting on carbon-nitrogen (but not peptide) bonds, in cyclic amidines | molecular_function | 0.018225 | 1 | 1 | 0 |
| GO:0042149 | cellular response to glucose starvation | biological_process | 0.018427 | 1 | 0 | 1 |
| GO:0006213 | pyrimidine nucleoside metabolic process | biological_process | 0.019309 | 1 | 1 | 0 |
| GO:0071158 | positive regulation of cell cycle arrest | biological_process | 0.019346 | 1 | 0 | 1 |
| GO:0006825 | copper ion transport | biological_process | 0.022479 | 1 | 0 | 1 |
| GO:0005227 | calcium activated cation channel activity | molecular_function | 0.022563 | 1 | 0 | 1 |
| GO:0001825 | blastocyst formation | biological_process | 0.02284 | 1 | 0 | 1 |
| GO:0000028 | ribosomal small subunit assembly | biological_process | 0.023939 | 1 | 0 | 1 |
| GO:0030686 | 90S preribosome | cellular_component | 0.024329 | 1 | 0 | 1 |
| GO:0030134 | ER to Golgi transport vesicle | cellular_component | 0.025085 | 1 | 0 | 1 |
| GO:0098781 | ncRNA transcription | biological_process | 0.025529 | 1 | 0 | 1 |
| GO:0010591 | regulation of lamellipodium assembly | biological_process | 0.026331 | 1 | 1 | 0 |
| GO:0000139 | Golgi membrane | cellular_component | 0.026633 | 2 | 1 | 1 |
| GO:0030173 | integral component of Golgi membrane | cellular_component | 0.028016 | 1 | 0 | 1 |
| GO:0071156 | regulation of cell cycle arrest | biological_process | 0.028832 | 1 | 0 | 1 |
| GO:0031228 | intrinsic component of Golgi membrane | cellular_component | 0.029206 | 1 | 0 | 1 |
| GO:0008138 | protein tyrosine/serine/threonine phosphatase activity | molecular_function | 0.029502 | 1 | 1 | 0 |
| GO:0005544 | calcium-dependent phospholipid binding | molecular_function | 0.030836 | 1 | 0 | 1 |
| GO:0022839 | ion gated channel activity | molecular_function | 0.032269 | 1 | 0 | 1 |
| GO:0071496 | cellular response to external stimulus | biological_process | 0.033219 | 2 | 1 | 1 |
| GO:0046915 | transition metal ion transmembrane transporter activity | molecular_function | 0.033405 | 1 | 0 | 1 |
| GO:0022613 | ribonucleoprotein complex biogenesis | biological_process | 0.035357 | 2 | 0 | 2 |
| GO:1902743 | regulation of lamellipodium organization | biological_process | 0.03564 | 1 | 1 | 0 |
| GO:0046854 | phosphatidylinositol phosphorylation | biological_process | 0.0361 | 1 | 1 | 0 |
| GO:0044452 | nucleolar part | cellular_component | 0.044051 | 1 | 0 | 1 |
| GO:0046834 | lipid phosphorylation | biological_process | 0.044626 | 1 | 1 | 0 |
| GO:0035064 | methylated histone binding | molecular_function | 0.044766 | 1 | 0 | 1 |
| GO:0072332 | intrinsic apoptotic signaling pathway by p53 class mediator | biological_process | 0.04563 | 1 | 0 | 1 |
| GO:0030032 | lamellipodium assembly | biological_process | 0.046466 | 1 | 1 | 0 |
| GO:0042255 | ribosome assembly | biological_process | 0.047724 | 1 | 0 | 1 |
| GO:0009405 | pathogenesis | biological_process | 0.047817 | 1 | 0 | 1 |
| GO:0005793 | endoplasmic reticulum-Golgi intermediate compartment | cellular_component | 0.047825 | 1 | 0 | 1 |
| GO:0000792 | heterochromatin | cellular_component | 0.048136 | 1 | 0 | 1 |
| GO:0001824 | blastocyst development | biological_process | 0.048511 | 1 | 0 | 1 |
| GO:0022857 | transmembrane transporter activity | molecular_function | 0.04874 | 3 | 0 | 3 |
