# Supplementary material for: The effects of resveratrol feeding and exercise training on the skeletal muscle function and transcriptome of aged rats
Source: PeerJ. 2019 Jul 1;7:e7199. doi: 10.7717/peerj.7199 (PMC6610545; doi:10.7717/peerj.7199)
Supplement: Table S3 — Old: old rat; Resveratrol: old rat treated by oral resveratrol; GO: Gene Ontology. [file peerj-07-7199-s003.doc]

Table S3 The significant Gene Ontology (GO) terms of the rats treated with six weeks of resveratrol feeding (Resveratrol) compared to the control rats (Old).

| GO accession | Description | Term type | Over represented p Value | DEG item | Up | Down |
| --- | --- | --- | --- | --- | --- | --- |
| GO:0031201 | SNARE complex | cellular_component | 2.35E-06 | 3 | 3 | 0 |
| GO:0000149 | SNARE binding | molecular_function | 2.37E-05 | 3 | 3 | 0 |
| GO:0008021 | synaptic vesicle | cellular_component | 3.31E-05 | 3 | 3 | 0 |
| GO:0017075 | syntaxin-1 binding | molecular_function | 4.45E-05 | 2 | 2 | 0 |
| GO:0006836 | neurotransmitter transport | biological_process | 7.01E-05 | 3 | 3 | 0 |
| GO:0001505 | regulation of neurotransmitter levels | biological_process | 7.68E-05 | 3 | 3 | 0 |
| GO:0060076 | excitatory synapse | cellular_component | 8.74E-05 | 2 | 2 | 0 |
| GO:0042572 | retinol metabolic process | biological_process | 8.95E-05 | 2 | 2 | 0 |
| GO:0098793 | presynapse | cellular_component | 0.000105 | 3 | 3 | 0 |
| GO:0034308 | primary alcohol metabolic process | biological_process | 0.000159 | 2 | 2 | 0 |
| GO:0048278 | vesicle docking | biological_process | 0.000258 | 2 | 2 | 0 |
| GO:0030672 | synaptic vesicle membrane | cellular_component | 0.00026 | 2 | 2 | 0 |
| GO:0016050 | vesicle organization | biological_process | 0.000291 | 3 | 3 | 0 |
| GO:0001523 | retinoid metabolic process | biological_process | 0.000298 | 2 | 2 | 0 |
| GO:0042734 | presynaptic membrane | cellular_component | 0.000312 | 2 | 2 | 0 |
| GO:0045202 | synapse | cellular_component | 0.000328 | 4 | 3 | 1 |
| GO:0006066 | alcohol metabolic process | biological_process | 0.000356 | 3 | 3 | 0 |
| GO:0016101 | diterpenoid metabolic process | biological_process | 0.000399 | 2 | 2 | 0 |
| GO:0022406 | membrane docking | biological_process | 0.000415 | 2 | 2 | 0 |
| GO:0006721 | terpenoid metabolic process | biological_process | 0.000528 | 2 | 2 | 0 |
| GO:0098746 | fast, calcium ion-dependent exocytosis of neurotransmitter | biological_process | 0.000575 | 1 | 1 | 0 |
| GO:0008068 | extracellular-glutamate-gated chloride channel activity | molecular_function | 0.000589 | 1 | 1 | 0 |
| GO:0042137 | sequestering of neurotransmitter | biological_process | 0.000589 | 1 | 1 | 0 |
| GO:0097401 | synaptic vesicle lumen acidification | biological_process | 0.000589 | 1 | 1 | 0 |
| GO:0035249 | synaptic transmission, glutamatergic | biological_process | 0.000637 | 2 | 2 | 0 |
| GO:0019905 | syntaxin binding | molecular_function | 0.000759 | 2 | 2 | 0 |
| GO:0034754 | cellular hormone metabolic process | biological_process | 0.000917 | 2 | 2 | 0 |
| GO:0006720 | isoprenoid metabolic process | biological_process | 0.001104 | 2 | 2 | 0 |
| GO:0030424 | axon | cellular_component | 0.001116 | 3 | 3 | 0 |
| GO:1901615 | organic hydroxy compound metabolic process | biological_process | 0.001214 | 3 | 3 | 0 |
| GO:0010975 | regulation of neuron projection development | biological_process | 0.0013 | 3 | 3 | 0 |
| GO:0007269 | neurotransmitter secretion | biological_process | 0.001609 | 2 | 2 | 0 |
| GO:0015319 | sodium:inorganic phosphate symporter activity | molecular_function | 0.001753 | 1 | 1 | 0 |
| GO:0042583 | chromaffin granule | cellular_component | 0.001775 | 1 | 1 | 0 |
| GO:0042584 | chromaffin granule membrane | cellular_component | 0.001775 | 1 | 1 | 0 |
| GO:0006906 | vesicle fusion | biological_process | 0.001989 | 2 | 2 | 0 |
| GO:0050806 | positive regulation of synaptic transmission | biological_process | 0.002058 | 2 | 2 | 0 |
| GO:0007270 | neuron-neuron synaptic transmission | biological_process | 0.00224 | 2 | 2 | 0 |
| GO:0030348 | syntaxin-3 binding | molecular_function | 0.002299 | 1 | 1 | 0 |
| GO:0070032 | synaptobrevin 2-SNAP-25-syntaxin-1a-complexin I complex | cellular_component | 0.002377 | 1 | 1 | 0 |
| GO:0007268 | synaptic transmission | biological_process | 0.002486 | 3 | 3 | 0 |
| GO:0044456 | synapse part | cellular_component | 0.002516 | 3 | 3 | 0 |
| GO:0090174 | organelle membrane fusion | biological_process | 0.002668 | 2 | 2 | 0 |
| GO:0031344 | regulation of cell projection organization | biological_process | 0.002675 | 3 | 3 | 0 |
| GO:0044300 | cerebellar mossy fiber | cellular_component | 0.002867 | 1 | 1 | 0 |
| GO:0042445 | hormone metabolic process | biological_process | 0.002873 | 2 | 2 | 0 |
| GO:0042904 | 9-cis-retinoic acid biosynthetic process | biological_process | 0.002889 | 1 | 1 | 0 |
| GO:0042905 | 9-cis-retinoic acid metabolic process | biological_process | 0.002889 | 1 | 1 | 0 |
| GO:0005315 | inorganic phosphate transmembrane transporter activity | molecular_function | 0.002928 | 1 | 1 | 0 |
| GO:0004028 | 3-chloroallyl aldehyde dehydrogenase activity | molecular_function | 0.002954 | 1 | 1 | 0 |
| GO:0045664 | regulation of neuron differentiation | biological_process | 0.003002 | 3 | 3 | 0 |
| GO:0002072 | optic cup morphogenesis involved in camera-type eye development | biological_process | 0.003368 | 1 | 1 | 0 |
| GO:0070050 | neuron cellular homeostasis | biological_process | 0.003505 | 1 | 1 | 0 |
| GO:0070324 | thyroid hormone binding | molecular_function | 0.003505 | 1 | 1 | 0 |
| GO:0048284 | organelle fusion | biological_process | 0.003643 | 2 | 2 | 0 |
| GO:0051938 | L-glutamate import | biological_process | 0.003955 | 1 | 1 | 0 |
| GO:0031915 | positive regulation of synaptic plasticity | biological_process | 0.003975 | 1 | 1 | 0 |
| GO:0044801 | single-organism membrane fusion | biological_process | 0.004021 | 2 | 2 | 0 |
| GO:0016188 | synaptic vesicle maturation | biological_process | 0.004032 | 1 | 1 | 0 |
| GO:0015321 | sodium-dependent phosphate transmembrane transporter activity | molecular_function | 0.004061 | 1 | 1 | 0 |
| GO:0005513 | detection of calcium ion | biological_process | 0.004524 | 1 | 1 | 0 |
| GO:0071705 | nitrogen compound transport | biological_process | 0.004602 | 3 | 3 | 0 |
| GO:0070327 | thyroid hormone transport | biological_process | 0.004611 | 1 | 1 | 0 |
| GO:0031340 | positive regulation of vesicle fusion | biological_process | 0.004632 | 1 | 1 | 0 |
| GO:0044341 | sodium-dependent phosphate transport | biological_process | 0.00464 | 1 | 1 | 0 |
| GO:0097060 | synaptic membrane | cellular_component | 0.004911 | 2 | 2 | 0 |
| GO:0031045 | dense core granule | cellular_component | 0.005204 | 1 | 1 | 0 |
| GO:0005436 | sodium:phosphate symporter activity | molecular_function | 0.00522 | 1 | 1 | 0 |
| GO:0002138 | retinoic acid biosynthetic process | biological_process | 0.005246 | 1 | 1 | 0 |
| GO:0016102 | diterpenoid biosynthetic process | biological_process | 0.005246 | 1 | 1 | 0 |
| GO:0061025 | membrane fusion | biological_process | 0.005272 | 2 | 2 | 0 |
| GO:0050767 | regulation of neurogenesis | biological_process | 0.005335 | 3 | 3 | 0 |
| GO:0060900 | embryonic camera-type eye formation | biological_process | 0.005549 | 1 | 1 | 0 |
| GO:0007611 | learning or memory | biological_process | 0.005939 | 2 | 2 | 0 |
| GO:2000060 | positive regulation of protein ubiquitination involved in ubiquitin-dependent protein catabolic process | biological_process | 0.006379 | 1 | 1 | 0 |
| GO:0000808 | origin recognition complex | cellular_component | 0.00639 | 1 | 1 | 0 |
| GO:0010976 | positive regulation of neuron projection development | biological_process | 0.007032 | 2 | 2 | 0 |
| GO:0051960 | regulation of nervous system development | biological_process | 0.007246 | 3 | 3 | 0 |
| GO:0016114 | terpenoid biosynthetic process | biological_process | 0.00739 | 1 | 1 | 0 |
| GO:0050890 | cognition | biological_process | 0.007439 | 2 | 2 | 0 |
| GO:0031175 | neuron projection development | biological_process | 0.007578 | 3 | 3 | 0 |
| GO:0043092 | L-amino acid import | biological_process | 0.007962 | 1 | 1 | 0 |
| GO:0016023 | cytoplasmic membrane-bounded vesicle | cellular_component | 0.00811 | 3 | 3 | 0 |
| GO:0015114 | phosphate ion transmembrane transporter activity | molecular_function | 0.008118 | 1 | 1 | 0 |
| GO:0035435 | phosphate ion transmembrane transport | biological_process | 0.008118 | 1 | 1 | 0 |
| GO:0031083 | BLOC-1 complex | cellular_component | 0.008333 | 1 | 1 | 0 |
| GO:0043090 | amino acid import | biological_process | 0.008497 | 1 | 1 | 0 |
| GO:0050750 | low-density lipoprotein particle receptor binding | molecular_function | 0.008545 | 1 | 1 | 0 |
| GO:0044433 | cytoplasmic vesicle part | cellular_component | 0.008756 | 2 | 2 | 0 |
| GO:0042403 | thyroid hormone metabolic process | biological_process | 0.009112 | 1 | 1 | 0 |
| GO:0044255 | cellular lipid metabolic process | biological_process | 0.009246 | 3 | 3 | 0 |
| GO:0043010 | camera-type eye development | biological_process | 0.009386 | 2 | 2 | 0 |
| GO:0060284 | regulation of cell development | biological_process | 0.009753 | 3 | 3 | 0 |
| GO:1903861 | positive regulation of dendrite extension | biological_process | 0.01001 | 1 | 1 | 0 |
| GO:0050804 | modulation of synaptic transmission | biological_process | 0.010069 | 2 | 2 | 0 |
| GO:0007267 | cell-cell signaling | biological_process | 0.010278 | 3 | 3 | 0 |
| GO:0005234 | extracellular-glutamate-gated ion channel activity | molecular_function | 0.010342 | 1 | 1 | 0 |
| GO:1901677 | phosphate transmembrane transporter activity | molecular_function | 0.010398 | 1 | 1 | 0 |
| GO:0042573 | retinoic acid metabolic process | biological_process | 0.010481 | 1 | 1 | 0 |
| GO:1903859 | regulation of dendrite extension | biological_process | 0.010595 | 1 | 1 | 0 |
| GO:0070325 | lipoprotein particle receptor binding | molecular_function | 0.010805 | 1 | 1 | 0 |
| GO:0072337 | modified amino acid transport | biological_process | 0.01093 | 1 | 1 | 0 |
| GO:0006817 | phosphate ion transport | biological_process | 0.010931 | 1 | 1 | 0 |
| GO:0031410 | cytoplasmic vesicle | cellular_component | 0.010976 | 3 | 3 | 0 |
| GO:0031082 | BLOC complex | cellular_component | 0.011199 | 1 | 1 | 0 |
| GO:0048488 | synaptic vesicle endocytosis | biological_process | 0.011221 | 1 | 1 | 0 |
| GO:0015813 | L-glutamate transport | biological_process | 0.011347 | 1 | 1 | 0 |
| GO:0031346 | positive regulation of cell projection organization | biological_process | 0.011494 | 2 | 2 | 0 |
| GO:0045956 | positive regulation of calcium ion-dependent exocytosis | biological_process | 0.0116 | 1 | 1 | 0 |
| GO:2000058 | regulation of protein ubiquitination involved in ubiquitin-dependent protein catabolic process | biological_process | 0.011612 | 1 | 1 | 0 |
| GO:0006144 | purine nucleobase metabolic process | biological_process | 0.011705 | 1 | 1 | 0 |
| GO:0043005 | neuron projection | cellular_component | 0.011786 | 3 | 3 | 0 |
| GO:0048666 | neuron development | biological_process | 0.011935 | 3 | 3 | 0 |
| GO:0045666 | positive regulation of neuron differentiation | biological_process | 0.012102 | 2 | 2 | 0 |
| GO:0048596 | embryonic camera-type eye morphogenesis | biological_process | 0.012325 | 1 | 1 | 0 |
| GO:0015800 | acidic amino acid transport | biological_process | 0.01246 | 1 | 1 | 0 |
| GO:0001654 | eye development | biological_process | 0.012524 | 2 | 2 | 0 |
| GO:0097484 | dendrite extension | biological_process | 0.012885 | 1 | 1 | 0 |
| GO:0048786 | presynaptic active zone | cellular_component | 0.012891 | 1 | 1 | 0 |
| GO:0014046 | dopamine secretion | biological_process | 0.01379 | 1 | 1 | 0 |
| GO:0014059 | regulation of dopamine secretion | biological_process | 0.01379 | 1 | 1 | 0 |
| GO:0036465 | synaptic vesicle recycling | biological_process | 0.014727 | 1 | 1 | 0 |
| GO:0051452 | intracellular pH reduction | biological_process | 0.015891 | 1 | 1 | 0 |
| GO:0065008 | regulation of biological quality | biological_process | 0.016243 | 5 | 5 | 0 |
| GO:0098796 | membrane protein complex | cellular_component | 0.016494 | 3 | 3 | 0 |
| GO:0048791 | calcium ion-dependent exocytosis of neurotransmitter | biological_process | 0.016609 | 1 | 1 | 0 |
| GO:0007616 | long-term memory | biological_process | 0.016671 | 1 | 1 | 0 |
| GO:0007399 | nervous system development | biological_process | 0.016677 | 4 | 4 | 0 |
| GO:0048048 | embryonic eye morphogenesis | biological_process | 0.016711 | 1 | 1 | 0 |
| GO:0045851 | pH reduction | biological_process | 0.016994 | 1 | 1 | 0 |
| GO:0001786 | phosphatidylserine binding | molecular_function | 0.01727 | 1 | 1 | 0 |
| GO:0023061 | signal release | biological_process | 0.017712 | 2 | 2 | 0 |
| GO:0031076 | embryonic camera-type eye development | biological_process | 0.018016 | 1 | 1 | 0 |
| GO:0005484 | SNAP receptor activity | molecular_function | 0.018224 | 1 | 1 | 0 |
| GO:0050769 | positive regulation of neurogenesis | biological_process | 0.018918 | 2 | 2 | 0 |
| GO:0015872 | dopamine transport | biological_process | 0.019595 | 1 | 1 | 0 |
| GO:0008299 | isoprenoid biosynthetic process | biological_process | 0.019703 | 1 | 1 | 0 |
| GO:0006629 | lipid metabolic process | biological_process | 0.019957 | 3 | 3 | 0 |
| GO:0044708 | single-organism behavior | biological_process | 0.020279 | 2 | 2 | 0 |
| GO:0046580 | negative regulation of Ras protein signal transduction | biological_process | 0.020634 | 1 | 1 | 0 |
| GO:0015296 | anion:cation symporter activity | molecular_function | 0.020652 | 1 | 1 | 0 |
| GO:0010817 | regulation of hormone levels | biological_process | 0.021009 | 2 | 2 | 0 |
| GO:0051058 | negative regulation of small GTPase mediated signal transduction | biological_process | 0.021104 | 1 | 1 | 0 |
| GO:0009112 | nucleobase metabolic process | biological_process | 0.021198 | 1 | 1 | 0 |
| GO:0016079 | synaptic vesicle exocytosis | biological_process | 0.021306 | 1 | 1 | 0 |
| GO:0030182 | neuron differentiation | biological_process | 0.021828 | 3 | 3 | 0 |
| GO:0097458 | neuron part | cellular_component | 0.021942 | 3 | 3 | 0 |
| GO:0030030 | cell projection organization | biological_process | 0.022169 | 3 | 3 | 0 |
| GO:0071702 | organic substance transport | biological_process | 0.022246 | 4 | 4 | 0 |
| GO:1903305 | regulation of regulated secretory pathway | biological_process | 0.023219 | 1 | 1 | 0 |
| GO:0071277 | cellular response to calcium ion | biological_process | 0.024018 | 1 | 1 | 0 |
| GO:0051962 | positive regulation of nervous system development | biological_process | 0.024132 | 2 | 2 | 0 |
| GO:0005544 | calcium-dependent phospholipid binding | molecular_function | 0.024684 | 1 | 1 | 0 |
| GO:0050433 | regulation of catecholamine secretion | biological_process | 0.024875 | 1 | 1 | 0 |
| GO:0051966 | regulation of synaptic transmission, glutamatergic | biological_process | 0.026111 | 1 | 1 | 0 |
| GO:0050432 | catecholamine secretion | biological_process | 0.026514 | 1 | 1 | 0 |
| GO:0046982 | protein heterodimerization activity | molecular_function | 0.026867 | 2 | 2 | 0 |
| GO:0010720 | positive regulation of cell development | biological_process | 0.027847 | 2 | 2 | 0 |
| GO:0005231 | excitatory extracellular ligand-gated ion channel activity | molecular_function | 0.02803 | 1 | 1 | 0 |
| GO:0003407 | neural retina development | biological_process | 0.028054 | 1 | 1 | 0 |
| GO:0031338 | regulation of vesicle fusion | biological_process | 0.028383 | 1 | 1 | 0 |
| GO:0005546 | phosphatidylinositol-4,5-bisphosphate binding | molecular_function | 0.028465 | 1 | 1 | 0 |
| GO:0048699 | generation of neurons | biological_process | 0.02875 | 3 | 3 | 0 |
| GO:0007423 | sensory organ development | biological_process | 0.028769 | 2 | 2 | 0 |
| GO:0030276 | clathrin binding | molecular_function | 0.029018 | 1 | 1 | 0 |
| GO:0030658 | transport vesicle membrane | cellular_component | 0.029033 | 1 | 1 | 0 |
| GO:0060291 | long-term synaptic potentiation | biological_process | 0.029597 | 1 | 1 | 0 |
| GO:0060079 | excitatory postsynaptic potential | biological_process | 0.03017 | 1 | 1 | 0 |
| GO:0048306 | calcium-dependent protein binding | molecular_function | 0.030818 | 1 | 1 | 0 |
| GO:0006820 | anion transport | biological_process | 0.031162 | 2 | 2 | 0 |
| GO:0046872 | metal ion binding | molecular_function | 0.031437 | 5 | 3 | 2 |
| GO:0072341 | modified amino acid binding | molecular_function | 0.031492 | 1 | 1 | 0 |
| GO:0051453 | regulation of intracellular pH | biological_process | 0.031854 | 1 | 1 | 0 |
| GO:0051937 | catecholamine transport | biological_process | 0.03225 | 1 | 1 | 0 |
| GO:0030667 | secretory granule membrane | cellular_component | 0.033871 | 1 | 1 | 0 |
| GO:0030641 | regulation of cellular pH | biological_process | 0.034077 | 1 | 1 | 0 |
| GO:0017158 | regulation of calcium ion-dependent exocytosis | biological_process | 0.034079 | 1 | 1 | 0 |
| GO:0043169 | cation binding | molecular_function | 0.034449 | 5 | 3 | 2 |
| GO:0022008 | neurogenesis | biological_process | 0.035205 | 3 | 3 | 0 |
| GO:0042562 | hormone binding | molecular_function | 0.035307 | 1 | 1 | 0 |
| GO:0043195 | terminal bouton | cellular_component | 0.035449 | 1 | 1 | 0 |
| GO:0006835 | dicarboxylic acid transport | biological_process | 0.035994 | 1 | 1 | 0 |
| GO:0016620 | oxidoreductase activity, acting on the aldehyde or oxo group of donors, NAD or NADP as acceptor | molecular_function | 0.036068 | 1 | 1 | 0 |
| GO:0015672 | monovalent inorganic cation transport | biological_process | 0.036257 | 2 | 2 | 0 |
| GO:0015807 | L-amino acid transport | biological_process | 0.036327 | 1 | 1 | 0 |
| GO:0005254 | chloride channel activity | molecular_function | 0.038284 | 1 | 1 | 0 |
| GO:0045595 | regulation of cell differentiation | biological_process | 0.039214 | 3 | 3 | 0 |
| GO:0005230 | extracellular ligand-gated ion channel activity | molecular_function | 0.039278 | 1 | 1 | 0 |
| GO:0051952 | regulation of amine transport | biological_process | 0.040005 | 1 | 1 | 0 |
| GO:0015844 | monoamine transport | biological_process | 0.040129 | 1 | 1 | 0 |
| GO:1902936 | phosphatidylinositol bisphosphate binding | molecular_function | 0.040531 | 1 | 1 | 0 |
| GO:0045055 | regulated secretory pathway | biological_process | 0.04074 | 1 | 1 | 0 |
| GO:0008306 | associative learning | biological_process | 0.040811 | 1 | 1 | 0 |
| GO:0016903 | oxidoreductase activity, acting on the aldehyde or oxo group of donors | molecular_function | 0.041096 | 1 | 1 | 0 |
| GO:0015837 | amine transport | biological_process | 0.04164 | 1 | 1 | 0 |
| GO:1902476 | chloride transmembrane transport | biological_process | 0.04181 | 1 | 1 | 0 |
| GO:0006885 | regulation of pH | biological_process | 0.042241 | 1 | 1 | 0 |
| GO:0030004 | cellular monovalent inorganic cation homeostasis | biological_process | 0.042336 | 1 | 1 | 0 |
| GO:0045921 | positive regulation of exocytosis | biological_process | 0.042655 | 1 | 1 | 0 |
| GO:0015108 | chloride transmembrane transporter activity | molecular_function | 0.043477 | 1 | 1 | 0 |
| GO:0005253 | anion channel activity | molecular_function | 0.043825 | 1 | 1 | 0 |
| GO:0015294 | solute:cation symporter activity | molecular_function | 0.0441 | 1 | 1 | 0 |
| GO:0060078 | regulation of postsynaptic membrane potential | biological_process | 0.044755 | 1 | 1 | 0 |
| GO:0006641 | triglyceride metabolic process | biological_process | 0.044924 | 1 | 1 | 0 |
| GO:0018958 | phenol-containing compound metabolic process | biological_process | 0.045165 | 1 | 1 | 0 |
| GO:0048489 | synaptic vesicle transport | biological_process | 0.045313 | 1 | 1 | 0 |
| GO:0097480 | establishment of synaptic vesicle localization | biological_process | 0.045313 | 1 | 1 | 0 |
| GO:0015696 | ammonium transport | biological_process | 0.045464 | 1 | 1 | 0 |
| GO:0048731 | system development | biological_process | 0.045489 | 5 | 5 | 0 |
| GO:0008203 | cholesterol metabolic process | biological_process | 0.045676 | 1 | 1 | 0 |
| GO:0017156 | calcium ion-dependent exocytosis | biological_process | 0.045762 | 1 | 1 | 0 |
| GO:0007420 | brain development | biological_process | 0.04719 | 2 | 2 | 0 |
| GO:1902652 | secondary alcohol metabolic process | biological_process | 0.047367 | 1 | 1 | 0 |
| GO:2000026 | regulation of multicellular organismal development | biological_process | 0.048285 | 3 | 3 | 0 |
| GO:0005509 | calcium ion binding | molecular_function | 0.048715 | 2 | 2 | 0 |
| GO:0097479 | synaptic vesicle localization | biological_process | 0.04909 | 1 | 1 | 0 |
| GO:0016125 | sterol metabolic process | biological_process | 0.049655 | 1 | 1 | 0 |
| GO:1903052 | positive regulation of proteolysis involved in cellular protein catabolic process | biological_process | 0.049725 | 1 | 1 | 0 |
| GO:0031398 | positive regulation of protein ubiquitination | biological_process | 0.049824 | 1 | 1 | 0 |
