# Supplementary material for: The effects of resveratrol feeding and exercise training on the skeletal muscle function and transcriptome of aged rats
Source: PeerJ. 2019 Jul 1;7:e7199. doi: 10.7717/peerj.7199 (PMC6610545; doi:10.7717/peerj.7199)
Supplement: Table S4 — Old: old rat; Trained: old rat treated by exercise training; Resveratrol: old rat treated by oral resveratrol. [file peerj-07-7199-s004.doc]

Table S4 The significant KEGG enriched pathways the rats treated with six weeks of exercise training and resveratrol feeding compared to the control rats.

| Pathway | KEGG ID | Input number | Background number | Corrected P-Value |
| --- | --- | --- | --- | --- |
| Trained vs Old up-regulated |  |  |  |  |
| Mucin type O-Glycan biosynthesis | rno00512 | 1 | 28 | 0.033 |
| Drug metabolism - other enzymes | rno00983 | 1 | 55 | 0.033 |
| Pyrimidine metabolism | rno00240 | 1 | 108 | 0.043 |
| Resveratrol vs Old up-regulated |  |  |  |  |
| Synaptic vesicle cycle | rno04721 | 3 | 63 | 0.000 |
| Nicotine addiction | rno05033 | 1 | 40 | 0.047 |
| Retinol metabolism | rno00830 | 1 | 80 | 0.047 |
| Insulin secretion | rno04911 | 1 | 86 | 0.047 |
| Retrograde endocannabinoid signaling | rno04723 | 1 | 103 | 0.047 |
| Glutamatergic synapse | rno04724 | 1 | 115 | 0.047 |
