# Supplementary figures and images for: The effects of resveratrol feeding and exercise training on the skeletal muscle function and transcriptome of aged rats
Source: PeerJ. 2019 Jul 1;7:e7199. doi: 10.7717/peerj.7199 (PMC6610545; doi:10.7717/peerj.7199)

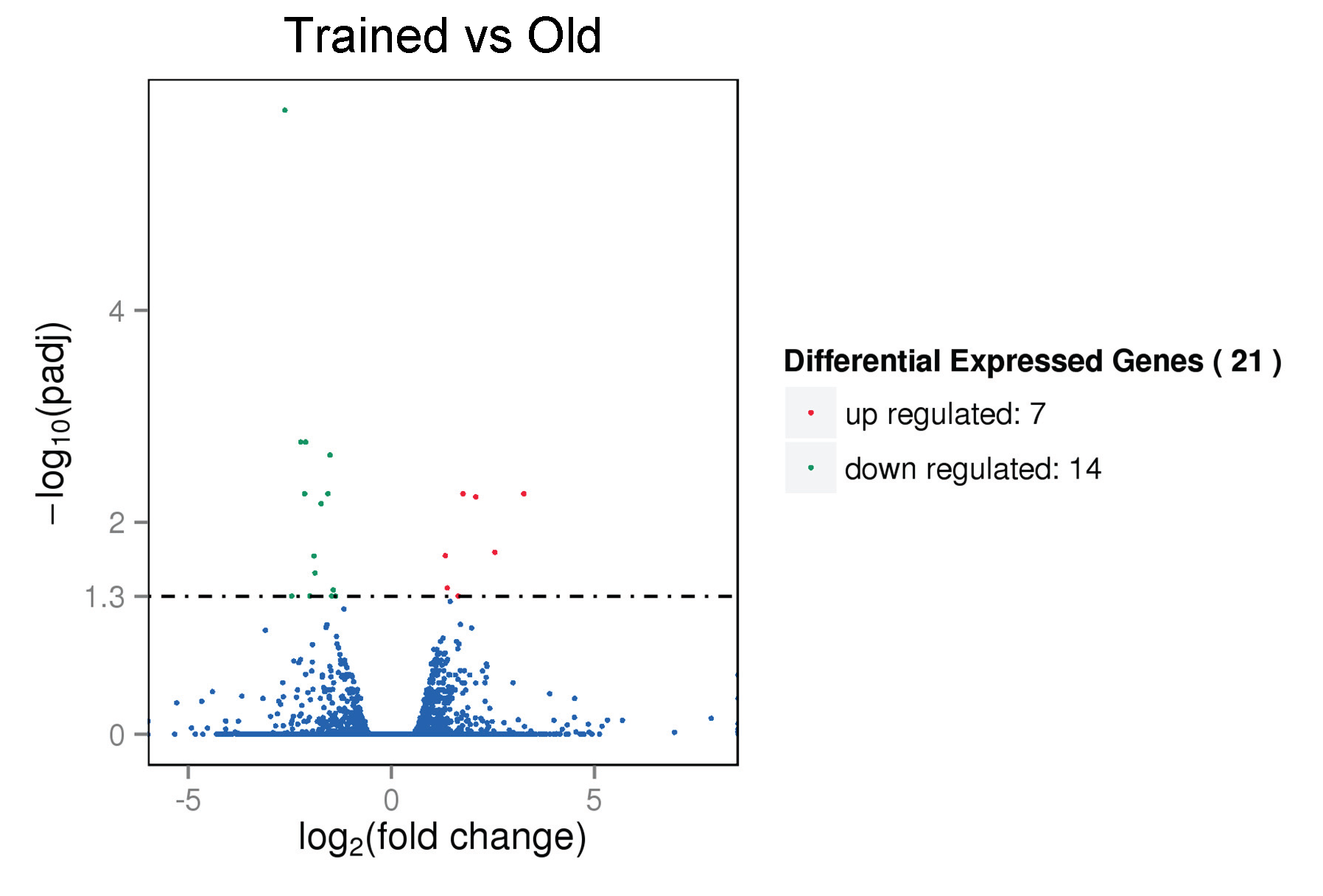

Supplement: Figure S1 — Old: old rat; Trained: old rat treated by six weeks of exercise training. [file peerj-07-7199-s005.png]

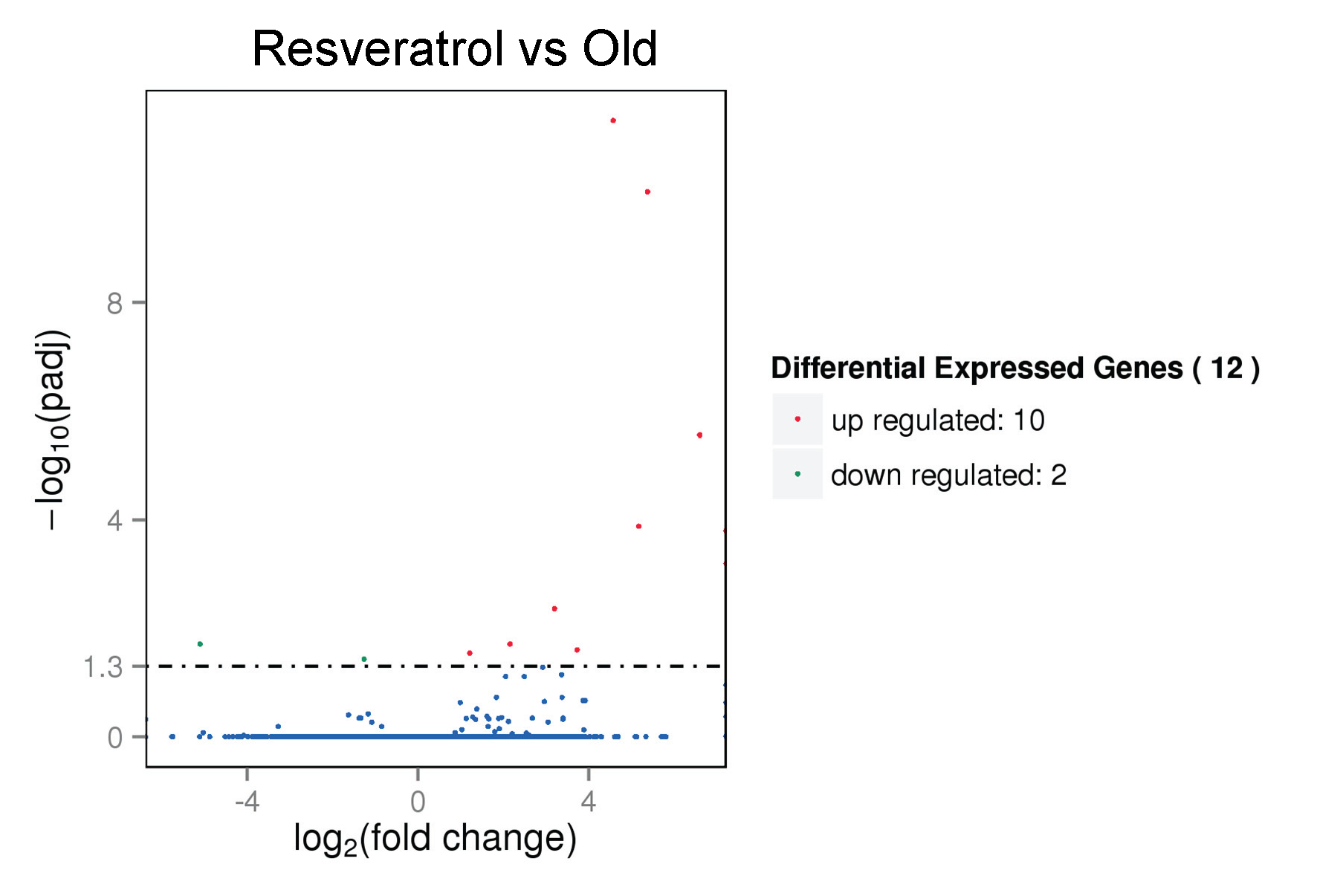

Supplement: Figure S2 — Old: old rat; Resveratrol: old rat treated by six weeks of oral resveratrol. [file peerj-07-7199-s006.png]
